# Diagnosis of rare tubular duplication small bowel malformation in adult woman by enteroscopy

**DOI:** 10.1055/a-2410-2183

**Published:** 2024-09-19

**Authors:** Jianyu Lv, Siyi Ni, Jianfen Wu, Shuo Zhang

**Affiliations:** 1 587400 Department of Gastroenterology, The Second Affiliated Hospital of Zhejiang Chinese Medical University, Hangzhou, China

Gastrointestinal duplication is rare, affecting males (78%) more than females 1 . The ileum is the most common site, accounting for over 60% of cases 2 . Duplications are mainly cystic (86%) or tubular 3 .

A 26-year-old woman with no medical history presented with intermittent abdominal pain. A previous colonoscopy suggested an ileocecal diverticulum. An abdominal computed tomography (CT) showed no issues ( Fig. 1 ). Small intestine CT showed no significant changes ( Fig. 2 ). We decided to perform a second colonoscopy and found an enteric structure with a double lumen next to the appendix opening ( Fig. 3 ).

**Fig. 1:**
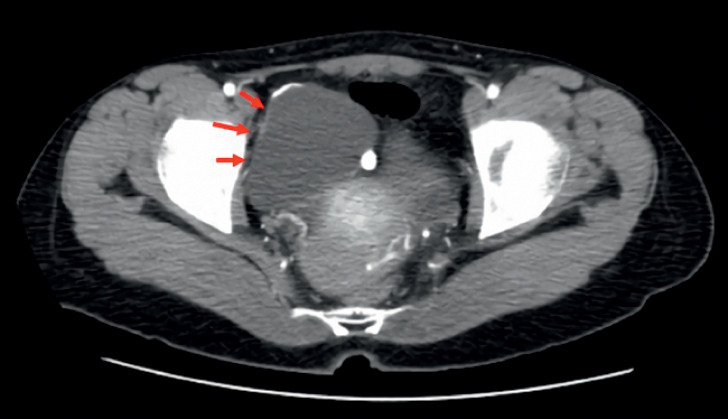
Abdominal enhanced computed tomography (CT), suggesting an irregular cystic low-density shadow on the right side of the pelvis.

**Fig. 2:**
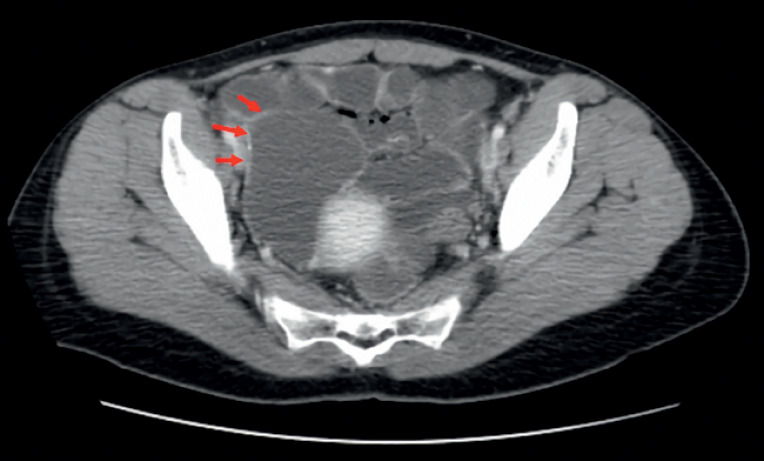
Small bowel CT enhancement, suggesting an irregular cystic lesion on the right side of the uterus, with no significant enhancement observed on enhanced scanning.

**Fig. 3:**
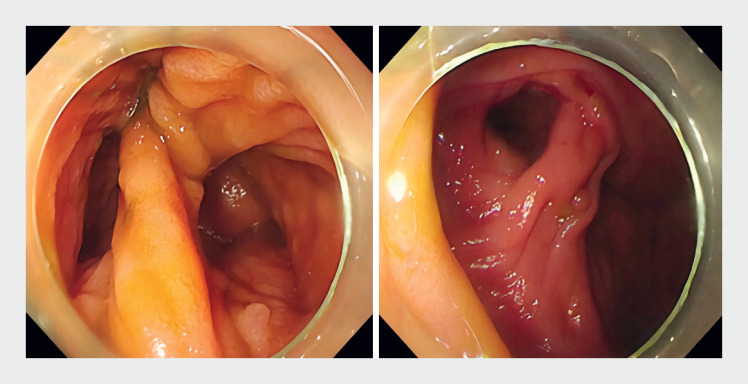
Endoscopic image showing a double-lumen small intestine.

To elucidate the anatomy, a small bowel endoscopy was performed, identifying a 3-cm ileocecal fistula with two distinct lumina mimicking small intestinal mucosa. Further exploration via the fistula exposed colonic mucosa. After retracting the endoscope and advancing it 20 cm into the small intestine, no abnormalities were observed. Titanium clips were placed at the fistula for orientation. Re-entry through the fistula confirmed one lumen with clips and the other leading to the ileocecal valve, indicative of intestinal duplication ( Fig. 4 , Fig. 5 , Video 1 ).

**Fig. 4:**
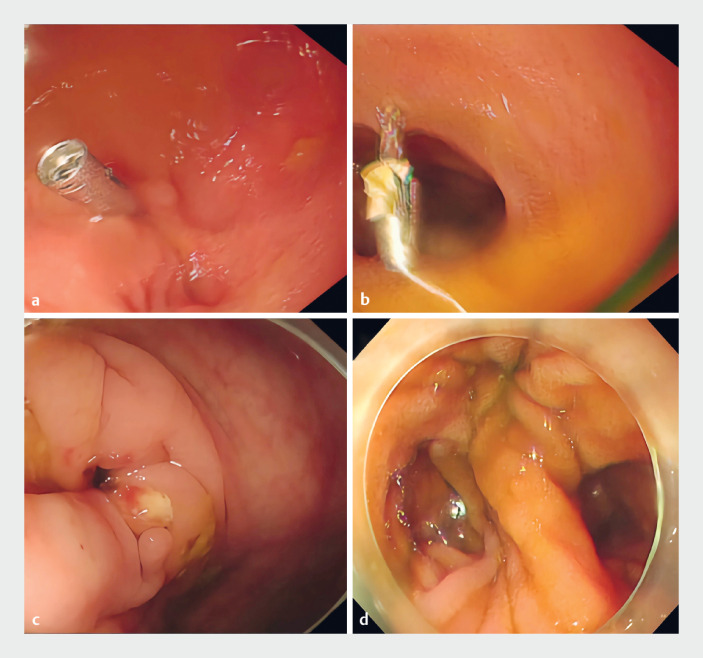
**a, b** Two titanium clips placed around the fistula opening, used to distinguish between the two intestinal lumina. **c** The ileocecal region could be reached through the intestinal lumen where no titanium clips were placed. **d** The double-lumen structure of the small intestine.

**Fig. 5:**
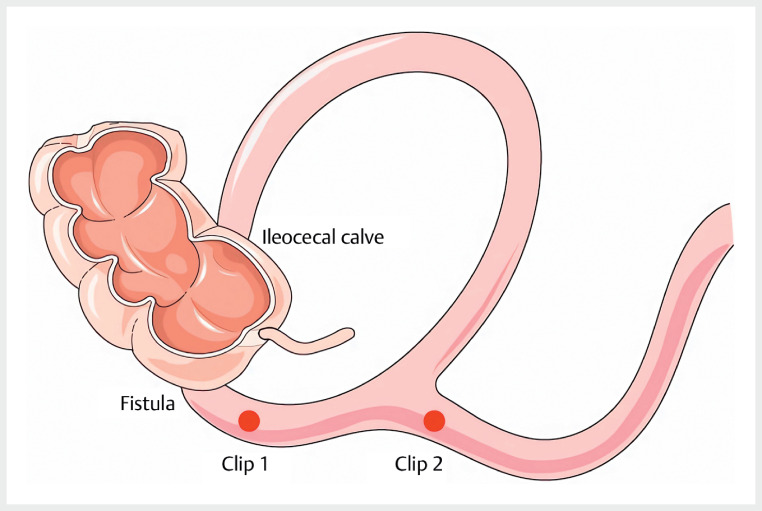
Schematic diagram of the intestinal duplication in this case. Source: modified according to Servier Medical Art under the license https://creativecommons.org/licenses/by/4.0/

Diagnosis of small intestine duplication with enteroscopy and capsule endoscopy.Video 1

An upper gastrointestinal (GI) contrast study revealed normal transit of barium through the stomach, duodenum, and jejunum. However, 1 hour post-administration, the patient was too unwell to cooperate. During the capsule endoscopy examination, we successfully re-located the titanium clips that were placed during the endoscopic procedure and confirmed the double-lumen structure of the small intestine, providing crucial evidence for the diagnosis of the case.

This case report successfully diagnosed a rare tubular duplication of the small bowel in an adult female using endoscopic titanium clip localization. Despite imaging studies not revealing significant abnormalities, endoscopy uncovered the lesion, demonstrating its unique value in identifying complex gastrointestinal pathologies. The article highlights the importance of endoscopy in diagnosing atypical symptoms and provides clinicians with new perspectives for managing similar cases.

Endoscopy_UCTN_Code_CCL_1AC_2AF

## Notes

Endoscopy E-Videos https://eref.thieme.de/e-videos
*E-Videos* is an open access online section of the journal *Endoscopy* , reporting on interesting cases and new techniques in gastroenterological endoscopy. All papers include a high-quality video and are published with a Creative Commons CC-BY license. Endoscopy E-Videos qualify for HINARI discounts and waivers and eligibility is automatically checked during the submission process. We grant 100% waivers to articles whose corresponding authors are based in Group A countries and 50% waivers to those who are based in Group B countries as classified by Research4Life (see: https://www.research4life.org/access/eligibility/ ). This section has its own submission website at https://mc.manuscriptcentral.com/e-videos .
